# Tracking drivers’ minds: Continuous evaluation of mental load and cognitive processing in a realistic driving simulator scenario by means of the EEG

**DOI:** 10.1016/j.heliyon.2023.e17904

**Published:** 2023-07-03

**Authors:** Edmund Wascher, Emad Alyan, Melanie Karthaus, Stephan Getzmann, Stefan Arnau, Julian Elias Reiser

**Affiliations:** IfADo - Leibniz Research Centre for Working Environment and Human Factors, Dortmund, Germany

**Keywords:** Driving, Mental load, EEG, Eye movements

## Abstract

Driving safety strongly depends on the driver's mental states and attention to the driving situation. Previous studies demonstrate a clear relationship between EEG measures and mental states, such as alertness and drowsiness, but often only map their mental state for a longer period of time. In this driving simulation study, we exploit the high temporal resolution of the EEG to capture fine-grained modulations in cognitive processes occurring before and after eye activity in the form of saccades, fixations, and eye blinks. A total of 15 subjects drove through an approximately 50-km course consisting of highway, country road, and urban passages. Based on the ratio of brain oscillatory alpha and theta activity, the total distance was classified into 10-m-long sections with low, medium, and high task loads. Blink-evoked and fixation-evoked event-related potentials, spectral perturbations, and lateralizations were analyzed as neuro-cognitive correlates of cognition and attention. Depending on EEG-based estimation of task load, these measures showed distinct patterns associated with driving behavior parameters such as speed and steering acceleration and represent a temporally highly resolved image of specific cognitive processes during driving. In future applications, combinations of these EEG measures could form the basis for driver warning systems which increase overall driving safety by considering rapid fluctuations in driver's attention and mental states.

## 1 Introduction

Driving a car is a complex task and requires continuous adaptation of attention based on the demands of the present driving situation. There is a long history of accessing the mental states of drivers in connection to drivers’ fatigue for a review see [1]. Among the neurophysiological methods used, EEG measures have turned out to be a valuable and promising tool to achieve this goal by measuring voltage changes directly on the scalp with high temporal precision without interfering with the task. Most studies, however, did not take advantage of this high temporal precision and concentrated on the tonic mental state of drivers over longer time periods.

However, this tonic driver state does not account for many of the multifaceted human factors that pose a risk for accidents. Besides phasic fluctuations of attention and distraction by irrelevant information or the operation of vehicle technology e.g. [[2], [3]], maladaptation to attentional demands of the driving situation (attentional effort) could endanger safe driving. Reliable and objectifiable methods to assess these states and processes could therefore significantly contribute to a better understanding of driver behavior and consequentially improve driving safety.

Another caveat of previous driving investigations is due to the trade-off between high experimental control and high ecological validity. Real-life behavior, such as driving a car in a natural situation, is not comparable to behavior in experimental situations in a laboratory, in which the systematic manipulation of safety-relevant factors is used to investigate cognitive processing. Experimental manipulation, for example, by controlled presentation of secondary task-related probing stimuli in a driving simulation, changes the driving situation in such a way that it no longer appears natural at its core. Thus, when addressing the investigation of mental processing in real-life behavior, the aim must be to assess mental states and processes in scenes that are as natural as possible.

Recently, we evaluated a possibility to investigate phasic changes in mental load using EEG measures. Analyzing the continuous spectral power changes of alpha and theta band activity in a naturalistic driving scene - using a driving simulator - provided valid [4] and reliable [5] indices for fluctuations in mental load with high temporal resolution. While this allows for the analysis of drivers' mental states depending on route sections of different difficulties, this still does not facilitate the investigation of event-specific cognitive processing, for example, when looking at a traffic sign on the roadside. Here, eye activity such as blinks and/or saccades was found to be very useful. By analyzing blink-evoked event-related potentials (bERPs) and blink-evoked event-related spectral perturbations (bERSPs) during natural tasks like walking, insights into visual processing, and visual and cognitive demands of natural behavior were provided without experimental manipulation of the (visual) scene [[6], [7], [8]]. This technique could also be applied to driving scenarios, although car driving may be more complex than walking in terms of eye behavior. During driving, mental processes comprise a dynamic allocation of attentional effort as well as an adaptation of covert and overt attention – once again stressing the importance of EEG measures with high temporal resolution. This interplay of covert and overt attention might be captured using extended eye-activity related analyses including horizontal eye movements. Relevant objects of the driving situation such as a sign on the side of the road might be detected in the periphery – by allocating covert attentional resources – before a saccade brings the object into the center of the view. After processing the detected object at that peripheral location, the gaze returns to the center of the road to process driving-relevant information from the visual scene facing the driving direction.

These oculomotor activities may serve as natural markers of information processing that allow for the investigation of mental processes and states during unrestricted driving. Several measures can be used to extract information about eye-activity related events. Firstly, *bERPs* have a similar waveform as regular event-related potentials (ERPs) following visual stimuli; positive and negative deflections after the event can be interpreted in terms of event-related changes in cognitive processing. This was shown in the laboratory [6] as well as in outside environments [7,9]. Changes in ERP components (namely P1, N1, P2, etc.) indicate modulations in sensory stimulus processing, attentional allocation as well as in the availability of mental resources [10]. Secondly, *fixation-evoked event-related potentials (fERPs)* have not only been investigated in laboratory-based visual search [11,12] and reading tasks [13], but also in natural environments [9,14]. Like bERPs, fERPs show a very similar morphology and experimental modulation compared to stimulus-evoked ERPs in the same task [15]. In general, event-related components could be extracted that imply fluctuations in cognitive processing. Additionally, the pronounced fERP P1 (also named lambda response [16]) is modulated by perceptual demands [14,17], and reduced with mental fatigue [16].

Besides these ERP modulations based on the emerging visual imagery generated after a blink or a new fixation, preparatory processes can also be assessed with respect to horizontal eye movements. Preceding a saccade, the subject's attention shifts towards the goal of the next fixation [premotor theory of attention; [18]. Thus, EEG activity preceding the saccade may also help to identify attentional behavior [19], for example, by the so-called event-related contingent negative variation (CNV). Additionally, event-related lateralizations (ERLs: the difference between contralateral and ipsilateral activations depending on the spatial code of a stimulus) of the EEG may serve as a marker for the spatial orienting of attention. Previous laboratory research showed that ERLs, such as increases in lateralized N1 amplitude or suppression in lateralized brain oscillatory alpha activity, could give insights into the shift of covert attention in a visual scenery [20], even in mobile situations [21].

The present analysis aimed at investigating to what extent these eye-event-related EEG parameters are suitable to represent fluctuations of (spatial) attention and mental driver load during a realistic driving task in a driving simulator. For this purpose, subjects drove through contiguous route profiles of different complexity and difficulty. It was expected that these modulations in route complexity and difficulty would be reflected in phasic and tonic parameters of neurocognitive correlates, indicating the cognitive processing of information along the route. Two stages of analyses were conducted: First, the task load along the route was estimated by means of fluctuations in EEG Theta and Alpha activity (as in our previous research, see Refs. [4,5]). For the estimation of task load, the ratio of Alpha and Theta power was taken as a basis, given that high Theta activity is associated with mental effort, whereas high Alpha activity coincides with reduced attentional allocation e.g. [[22], [23], [24]]. Based on this EEG-based estimation of task load, parameters of driving performance and eye activity-related potentials within sections of a given mental load were studied. By exploiting not only eye-blink behavior, but also fixation and saccade activity, we aimed to uncover subtle changes in cognitive processing and attentional allocation to a dynamic driving environment without adding extra events that could potentially compromise an immersive driving experience.

## 2 Methods

### 2.1 Participants

In this study, we recruited 20 participants without any prior or present neurologic or psychiatric condition, using online announcements and postings on bulletin boards at the Technical University Dortmund, Germany. All participants had no motor impairment, had a normal or corrected-to-normal vision, and were right-handed. Also, they had a driver's license for at least 3 years. Due to technical issues, only 15 datasets could be used for further analysis. The participants' age ranged from 20 to 26 years (mean = 22.53, standard deviation = 2.13), 6 were female, 9 male. The study was approved by the local ethics committee of the Leibniz Research Centre for Working Environment and Human Factors (March 24, 2017) and all participants gave written informed consent.

### 2.2 Task and procedure

**Fig. 1:**
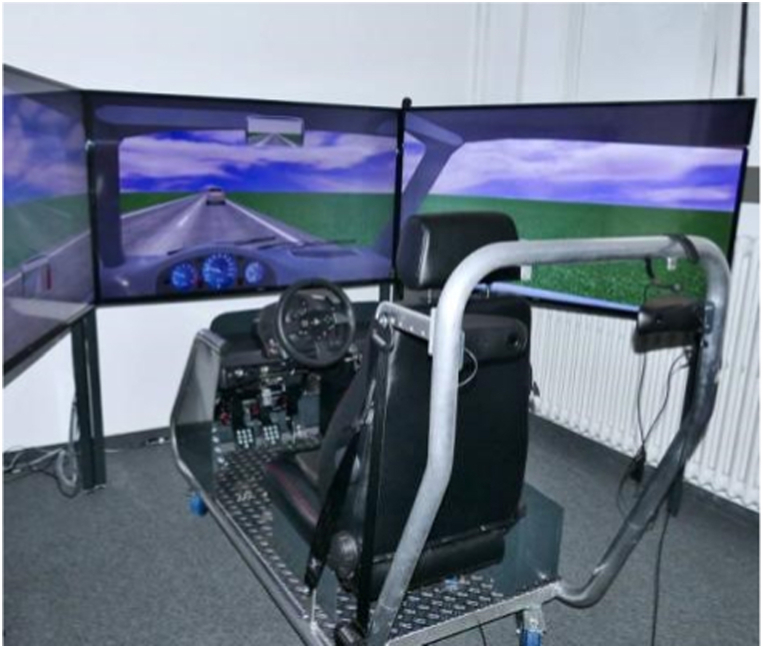
ST-Stim Driving simulator with a custom-made cockpit mock-up and three displays. An EEG-amplifier was situated behind the head rest.

When participants arrived at the lab around 9–10 a.m., they were fitted with a 10–20 system 32 electrode cap (actiCap, Brain Products GmbH, Gilching, GER). Participants then read the experimental instructions, gave their informed consent, and filled out questionnaires about demographic data, driving history and driving habits. After preparation, participants executed several conditions in a cognitive-motor dual-task paradigm which will not be reported here in detail. Right after this task, participants were escorted to the driving simulator laboratory, where they performed the driving task. This task consisted of a longer car ride with different road sections and typical driving situations of a German street environment. The ride took place in a static driving simulator (ST Sim, St Software B.V. Groningen, NL, see Fig. 1).

After a 5–10-min practice drive to familiarize the participants with the driving simulator, the route started with a two-lane freeway section. In this study, the focus lay on driving situations in which conspicuities in driving performance are typically observed, such as intersections, turning maneuvers (especially left turns), lane changes, and situations that require interaction with other road users. The following scenarios were implemented along the entire route: 52 intersections (9 with a left turn, 19 with a right turn, and 24 going straight; 5 with traffic lights; 4 with a stop sign; a total of 28 with right of way), 2 roundabouts, 34 speed limits (15 urban and 19 extra-urban), 2 freeway entrances and exits, 9 sections requiring evasive maneuvers (road works, obstacles, 5 extra-urban and 4 urban), 2 overtaking passages (optional) and 1 passage with prohibited overtaking, 1 foggy passage, as well as other critical events (including obstacles on the roadway, crosswalk with pedestrians on the roadway, bus stops, traffic-calmed zone). After completing the initial freeway section, participants entered a rural road, before driving back to the highway. After another section on the rural road, they finally drove into the city. The route had an average to upscale requirement character, comparable to that of an official driving test as estimated by an experienced driving instructor, with an estimated duration of 60 min.

Acoustic and visual navigation signals guided the drivers and indicated upcoming turn maneuvers. These consisted of spoken instructions from a female German speaker and the simultaneous presentation of directional arrows on a dashboard screen. If participants did not comply with the instructions, they were re-routed to a section of the track just before the turn. The participants were instructed to keep to the speed limit. The entire trip was about 51 km long and took the participant between 36 and 50 min to complete the first 50 km, the section that was analyzed.

After both the practice drive and the test drive, participants filled out a Motion Sickness Questionnaire [25] to register any account of discomfort due to exposure to the virtual environment.

### 2.3 Data recording

Electrophysiological data were recorded using a regular 10-20-system electrode cap (actiCap) that was fitted over the head of the participant which was equipped with 32 active electrodes (Fp1, Fp2, F3, F4, F7, F8, Fz, FC1, FC2, FC5, FC6, C3, C4, Cz, T7, T8, CP1, CP2, CP5, CP6, P3, P4, P7, P8, Pz, PO9, PO10, O1, O2, Oz). After aligning the cap properly, the electrodes were filled with electrolyte gel until an impedance of ≤10 kΩ was reached to ensure proper signal quality. The electrode cables were carefully aligned not to cross or to move relative to each other in order to prevent electromagnetic noise. The cables were then routed through loopholes near the participant's ear and plugged into a LiveAmp 32 amplifier (BrainProducts GmbH, Gilching, GER) placed in the actiCap's pocket at the back of the participant's head. Data were recorded with a sampling rate of 500 Hz and a bit depth of 24 bits directly onto a micro-SD card inserted into the amplifier. The EEG data were stored together with data from the driving simulator (speed and position of the steering wheel).

### 2.4 Data processing

#### 2.4.1 EEG-preprocessing

Data were initially band-pass filtered from 0.1 to 40 Hz and then entered into the PrepPipeline [26]. This function detects corrupt channels based on iterative procedures leading to a robust average reference and interpolates artificial channels. These data were high-pass filtered at 1 Hz and down-sampled to 250 Hz for ICA decomposition. Data were segmented into epochs of 1s length and checked for artifacts. Before entered into an ICA [27] data were compressed using PCA to the rank corresponding to the number of non-corrupt channels minus 1. Obtained ICs were categorized using ICLabel [28]. IC-weights were then written back to the initially 0.1Hz filtered data with a 500Hz sampling rate.

Based on ICA decomposition, eye blinks were identified by gaussian fits [7]. Saccades were detected by searching for maximal velocity in the IC identified to reflect horizontal eye movements. From this first estimation of possible saccades, templates (visually checked for a correct saccade shape) were determined for 3 expressions and correlated with the time courses in the ICs. Only segments with a correlation higher than 0.8 were specified in more detail and used for further analysis. For each saccade, both the onset and the offset, which reflect a new fixation, were determined. For further analyses, each saccade was additionally labelled for its direction (left/right) and whether it was directed outwards (away from a central fixation) or inwards (towards a central fixation). This factor was labelled “type of saccades”. For EEG analyses, ICs that were classified by ICLabel as brain activity with a probability of ≤ 30% were removed.

#### 2.4.2 Track-based determination of mental load

All data were intended to be analyzed in a scene-based manner [4]. Therefore, the car's exact position at any time point was calculated based on the directly transferred velocity and 85 predefined trigger points. The latter were moments of distinct events with a known position on the track. Since velocity and steering angle were transcribed to the EEG system only with a sampling rate of 100 Hz, moments of acceleration or breaking may bias the exact calculation of waypoints based solely on these data. This error was corrected by morphing the exact distance values between two trigger points. Finally, the entire track was divided into segments of 10 m length based on the distance covered by the driver, resulting in 5000 segments overall. For further analyses, each time point was assigned to these segments.

Then, EEG-data were entered into a continuous wavelet transform from 2 to 30 Hz in 29 steps separately for all EEG channels. Each trace was z-transformed for the entire time and averaged for the 5000 segments of 10-m length [4]. The outcome of this procedure was smoothed by a moving average ( ± 50 m), before Theta power for a frontal cluster (F3, Fz, F4, FC1, FC2) and Alpha power for a posterior cluster (P3, Pz, P4, PO9, PO10, O1, Oz, O2) were extracted. For the EEG-based estimation of task load, the ratio of Alpha and Theta power was used: For each subject and each segment, the difference between these two measures was calculated before the median across subjects of these difference values was calculated for each segment. These median-values were divided into terciles, assigning each segment either low (predominant Alpha power), medium (quite similar Alpha and Theta power), or high task load (predominant Theta power).

#### 2.4.3 Behavioral data

Average driving velocity, steering acceleration, as well as the frequency of blinks and saccades, were determined for each segment. Average values were calculated for each of these measures and for each level of mental load.

### 2.5 EEG data

#### 2.5.1 Eye-event-evoked EEG activity

For all eye-event-related analyses, eye events (blinks, saccade onsets and fixations) were labelled according to the determined task load of the segment in which they appeared. For a list of all analyzed eye-event-related EEG activity, see Table 1.

**Table 1:** Extracted electrophysiological measures with related eye activity, time of appearance, and EEG electrode sides.

Measure	Eye activity	Timing (ms)	Sites
N1	blink, fixation	[20, 200] [20, 200]	Occipital Occipital
P2	blink, fixation	[80, 200], [80, 250] [100, 300], [100, 400]	Parietal, occipital Parietal, occipital
N2	blink, fixation	[100, 600] [150, 400]	Fronto-central Fronto-central
N1pc	fixation	[80, 120]	Posterior
Tonic ERL	saccade	[-400, −200], [200, −50]	Posterior, posterior
CNV	saccade	[-200, −50]	Fronto-central
Alpha	saccade	[-800, −50]	Posterior

For eye-blink-related activity, data were selected time-locked to the center of the fitted Gaussian function that was used to detect blinks (which more or less accords to the maximum deflection in the time course of the eye-blink-related IC). Segments from −500 ms to 1000 ms were extracted. The baseline was set before blink onset (−400 to −200 ms with respect to the maximum). In the averaged ERPs, peak amplitudes were estimated using a jackknife procedure [29] for an occipital N1 (detection window: 20 to 200 ms; electrode cluster: O1, Oz, O2), a parietal P2 (80 to 200 ms, electrode cluster: CP1, CP2, P3, Pz, P4), an occipital P2 (80 to 250 ms) and a fronto-central N2 (detection window: 100 to 600 ms, electrode cluster: Cz, FC1, FC2, Fz).

The fixation-related activity was also analyzed in ERPs from −500 to 1000 ms with a baseline during saccade occlusion (−200 to 0 ms). The same electrode clusters as above were used. The occipital lambda response was determined between 0 and 200 ms, the occipital N1 between 20 and 200 ms, the parietal P2 between 100 and 300 ms, the occipital P2 between 100 and 400 ms, and finally the fronto-central N2 between 150 and 400 ms.

#### 2.5.2 (Spatial) attention-related EEG activity

As a phasic ERL component, the fixation-related asymmetry in the N1 range was determined as the mean amplitude of asymmetry in the contra-ispi difference waves between 80 and 120 ms after fixation, in a posterior electrode cluster (P7/P8, PO9/PO10, O1/O2). The fronto-central CNV preceding saccade onset was measured as the mean amplitude between −200 and −50 ms in the above defined electrode cluster. As another sustained (slow) component with additional spatial aspects, the tonic ERL was measured in two time windows at posterior sites preceding the onset of the saccade (−400 to −200 ms and −200 to −50 ms). All parameters here were entered into ANOVAs with the factors task load and type of saccade.

Event related lateralizations of saccade-related EEG activity were also investigated with time-frequency analysis by calculating the event-related spectral perturbations (ERSPs) using 29 frequency traces between 2 and 30 Hz obtained from complex Morlet wavelet convolution.

### 2.6 Statistical analysis

Regarding the behavioral measures, the average values for driving velocity, steering acceleration, and the average frequency of blinks and saccades were entered into a repeated-measures analyses of variance (ANOVAs) with the within-subjects factor task load (low, medium, high). Additionally, the factor type of saccade (outwards vs. inwards) was analyzed for saccade frequency. The blink-related ERPs, that is the occipital, the parietal P2, the occipital P2, and the fronto-central N2 were analyzed using an ANOVA with the within-subjects factor task-load. Fixation-related EEG activity were analyzed using an ANOVA with the factors task-load and saccade-type (inwards versus outwards). This applies to the occipital lambda response, the occipital N1, the parietal P2, the occipital P2, and the fronto-central N2. Preceeding the saccades, the frontal CNV, as well as the posterior lateralizations were analyzed the same way.

All *p*-values obtained from ANOVAs were Greenhouse-Geisser corrected when indicated. As an indicator of effect size, adjusted partial eta squared (adj *η*_p_^2^ [30]; are presented. To keep the manuscript concise, effect sizes are only reported for significant effects.

Regarding the results from the time-frequency analysis of EEG data time-locked to the saccades, the asymmetries in the EEG were investigated by calculating the ERSP for contra- and ipsilateral activity. Then, a cluster-based permutation test was performed on data averaged across all task load conditions to test the ERSP for ipsi- and contralateral activity against zero. The test used 1000 permutations and a clustering threshold of t-value corresponding to a p-value of p = .01, and a significance level for the cluster test statistic of *p* = .05. Since a distinct alpha suppression contralateral to saccade direction was observed (at least for inwards saccades) at PO7, alpha-power for this electrode between −800 and −50 ms was extracted and entered in an ANOVA with the same factors as all analyses described above.

## 3 Results

### 3.1 Behavioral data

**Fig. 2:**
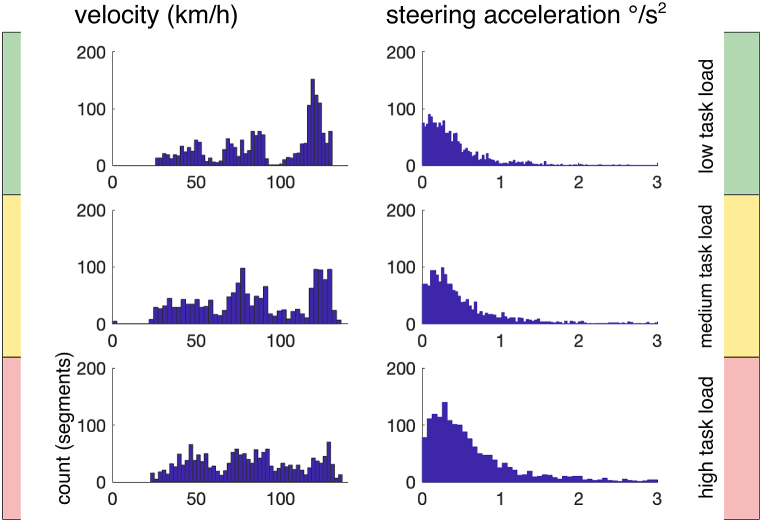
Histograms of driving parameters (number of segments), shown separately for the three levels of EEG-based estimation of task load. Note that all types of roads (freeway, state road, city) can appear in each level of task load. Segments with high velocity (freeway) are more often estimated as low task load, while segments with more steering acceleration are more probably estimated as high task load.

Average driving velocity decreased with increasing task load, *F*(2,28) = 304.04, *p* < .001, adj *η*_p_^2^ = 0.95. Steering acceleration showed the inverse effect and increased with task load, *F*(2,28) = 180.06, *p* < .001, adj *η*_p_^2^ = 0.92 (see Fig. 2 and Table 2).

**Table 2:** Means (and standard errors of means) for behavioral parameters, separated for the three levels of task load. Velocity decreased with increasing task load and steering acceleration increased. Blink frequency became lower and saccade frequency higher with task load.

	Low	Medium	High
Driving parameters			
Velocity (km/h)	91.37 (1.53)	82.792 (1.32)	78.924 (1.32)
Steering (°/s^2^)	6.00 (0.47)	9.38 (0.68)	12.70 (0.87)
Eye activity parameters			
Blinks/min	11.00 (0.62)	9.51 (0.51)	8.64 (0.62)
Saccades/min			
outwards	14.61 (0.69)	16.56 (0.39)	16.55 (0.50)
inwards	16.61 (0.73)	17.23 (0.54)	17.08 (0.60)

Blink frequency (see Table 2) decreased with increasing task load, *F*(2, 28) = 30.00, *p* < .001, adj *η*_p_^2^ = 0.66. In contrast, saccade frequency slightly increased, *F*(2,28) = 5.23, *p* = .030, adj *η*_p_^2^ = 0.22. More inwards than outwards saccades were performed, *F*(1,14) = 9.77, *p* = .007, adj *η*_p_^2^ = 0.37. The interaction between task load and saccade type, *F*(2,28) = 5.47, *p* = .011, adj *η*_p_^2^ = 0.23, indicates that the type effect was most pronounced in easy sections. Testing the task load effect separately for the two saccade types, we found a significant effect of task load for outward saccades, *F*(2,28) = 7.53, *p* = .009, adj *η*_p_^2^ = 0.30, but not for inward saccades, *F*(2,28) = 1.15, *p* > .2.

### 3.2 EEG data

#### 3.2.1 Onset related activity

Assuming that both, the re-opening of the eye during a blink and the fixation of a new object denotes moments of a new visual impression, we analyzed ERPs time-locked to these events in a comparable fashion.

#### 3.2.2 Blink-evoked ERPs

**Fig. 3:**
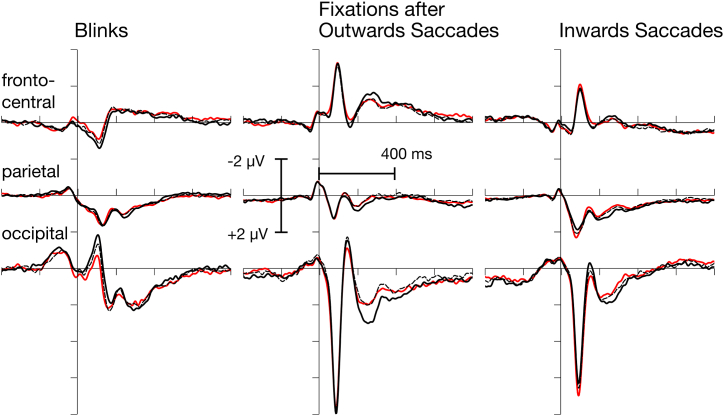
Event-related potentials (ERPs) evoked by vision-onset eye events for fronto-central, parietal and occipital electrode clusters, superposed for the three levels of task load (low: black solid; medium: black dashed; high: red). For blink-evoked potentials, only the reduction of the occipital N1 with increasing task load reached significance. Fixation-related potentials were generally larger for outward saccades. Task load effects were observed for the P1 and the occipital P2 evoked by inward saccades. A main effect of task load was found for the occipital N1 and the fronto-central N2. (For interpretation of the references to color in this figure legend, the reader is referred to the Web version of this article.)

N1 amplitude decreased (see Fig. 3, left column and Table 3) with task load, *F*(2,28) = 6.19, *p* = .0150, adj *η*_p_^2^ = 0.26. All other component amplitudes did not vary with task load (P2_parietal_: *F*(2,28) = 0.26, P2_occipital_: *F*(2,28) = 1.62, N2: *F*(2,28) = 0.47, all *ps* > .2).

**Table 3:** Means (and standard errors of means) in μV for the three levels of task load for all eye-event-related onset components derived from the ERPs.

	Low	Medium	High			
	Blinks					
N1	−1.72 (0.77)	−1.20 (0.60)	−0.62 (0.57)			
P2_parietal_	1.61 (0.31)	1.65 (0.33)	1.58 (0.33)			
P2_occipital_	2.26 (0.17)	2.07 (0.24)	1.97 (0.18)			
N2	−0.64 (0.33)	−0.83 (0.33)	−0.73 (0.22)			
Fixations						
	outwards			inwards		
P1	7.51 (1.01)	7.22 (0.98)	7.25 (0.94)	5.98 (0.71)	6.30 (0.82)	6.67 (0.78)
N1	−1.45 (0.55)	−1.58 (0.50)	−1.02 (0.44)	−0.21 (0.64)	0.21 (0.64)	0.49 (0.58)
P2_parietal_	0.82 (0.14)	0.58 (0.14)	0.59 (0.15)	1.60 (0.26)	1.81 (0.25)	1.72 (0.27)
P2_occipital_	4.66 (0.74)	4.36 (0.73)	4.46 (0.70)	4.74 (0.61)	5.22 (0.68)	5.41 (0.67)
N2	−1.65 (0.20)	−1.28 (0.15)	−1.26 (0.20)	−0.39 (0.18)	−0.21 (0.19)	−0.21 (0.17)

### 3.3 Fixation-evoked ERPs

The initial P1 (lambda response) did overall not vary with task load, *F*(2,28) = 1.44, *p* > .2, but was larger for outwards saccades, *F*(1,14) = 10.35, *p* = .006, adj *η*_p_^2^ = 0.38. Additionally, an interaction of task load by saccade types was observed, *F*(2,28) = 5.84, *p* = .012, adj *η*_p_^2^ = 0.24. A task load effect was not observed for outwards saccades, *F*(2,28) = 1.67, *p* > .2, but for inwards ones, *F*(2,28) = 4.88, *p* = .02, adj *η*_p_^2^ = 0.21 (for fixation evoked ERPs see Fig. 3, middle and right column, and Table 3).

As for blinks, N1 decreased with increasing task load, *F*(2,28) = 6.04, *p* = .010, adj *η*_p_^2^ = 0.25. Also, this component was larger for outward saccades, *F*(1,14) = 17.97, *p* < .001, adj *η*_p_^2^ = 0.53. No interaction between these two factors was observed, *F*(2,28) = 2.21, *p* = .13. The P2 at parietal sites did not vary with task load, *F*(2,28) = 0.30, *p* > .2, but with type of saccades, *F*(1,14) = 15.62, *p* = .001, adj *η*_p_^2^ = 0.49. The latter effect was modulated by task load, *F*(2,28) = 6.93, *p* = .005, adj *η*_p_^2^ = 0.28. Amplitude tended to decrease with task load for outwards saccades, *F*(2,28) = 3.22, *p* = .084, adj *η*_p_^2^ = 0.13, but to increase with inwards saccades, *F*(2,28) = 2.98, *p* = .082, adj *η*_p_^2^ = 0.12. Similar effects were observed for the occipital P2. No main effect of task load, *F*(2,28) = 1.22, *p* > .2, but a marginal effect of saccade type, *F*(1,14) = 3.53, *p* = .081, adj *η*_p_^2^ = 0.14, was overserved. Again, an interaction of task load by type of saccade was found, *F*(2,28) = 7.91, *p* = .002, adj *η*_p_^2^ = 0.32. No effect was found for outwards saccades, *F*(2,28) = 1.57, *p* > .2, but a reliable increase of this component with task load for inward saccades, *F*(2,28) = 5.28, *p* = .014, adj *η*_p_^2^ = 0.22.

Finally, the fronto-central N2 decreased in amplitude with increasing task load, *F*(2,28) = 6.00, *p* = .007, adj *η*_p_^2^ = 0.25. It was also substantially larger for outwards saccades, *F*(1,14) = 22.34, *p* < .001, adj *η*_p_^2^ = 0.59. There was no interaction, *F*(2,28) = 0.61, *p* > .2.

#### 3.3.1 (Spatial) attention

**Fig. 4:**
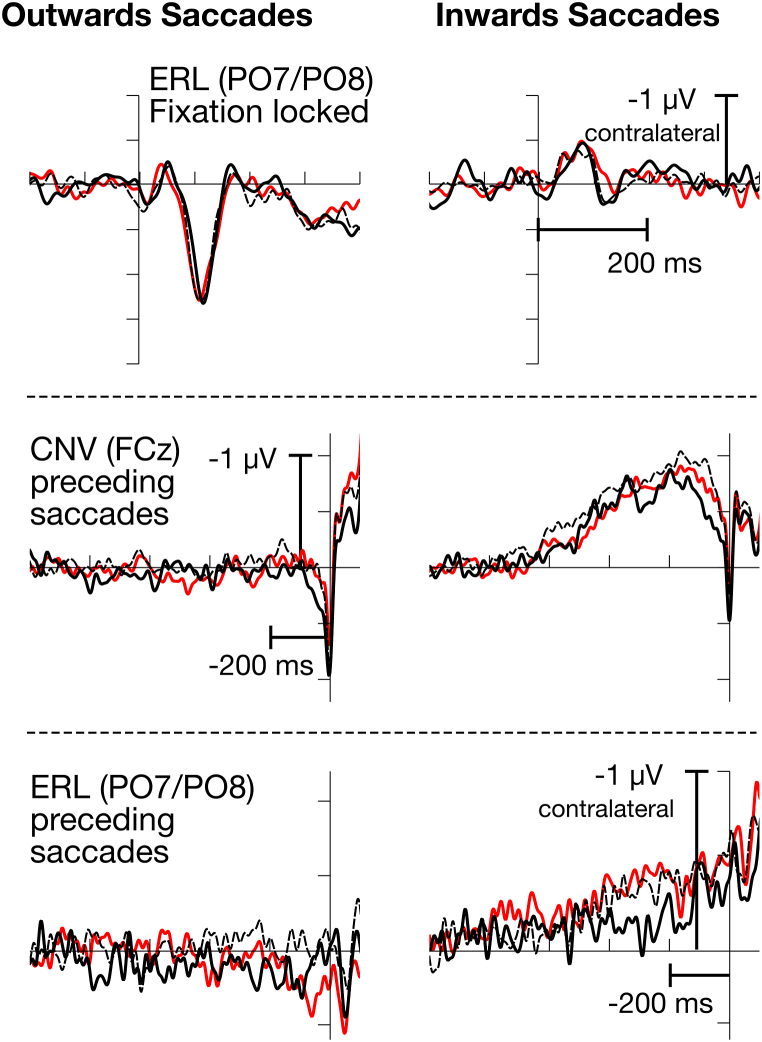
Fixation locked N2pcs (upper row), saccades preceding CNVs (middle row), and saccades preceding ERLs (lower row), superposed for the three levels of task load (low: black solid; medium: black dashed; high: red). The N2pc was substantially larger for outward than for inward saccades, while the CNV and ERL were found to be larger for inward saccades. (For interpretation of the references to color in this figure legend, the reader is referred to the Web version of this article.)

Not only centrally evoked ERPs are of interest in the context of saccades, but also spatial components, since fixations follow either a leftward or a rightward eye movement. Based on this, event-related lateralizations of the EEG can be calculated (see Fig. 4, 1st and 3rd row and Table 4) defined by the difference between contra- and ipsilateral activity with respect to the spatial position of a stimulus.

**Table 4:** Means (and standard errors of means) for measures of attention-related EEG activity.

	Low	Medium	High			
	outwards			inwards		
Fixation related						
phasic ERL	0.82 (0.26)	0.99 (0.24)	1.00 (0.27)	−0.11 (0.15)	−0.14 (0.13)	−0.30 (0.20)
Saccade related						
CNV	0.07 (0.10)	−0.09 (0.10)	−0.06 (0.08)	−0.66 (0.15)	−0.93 (0.12)	−0.81 (0.16)
Tonic ERL1	0.15 (0.11)	−0.06 (0.07)	0.13 (0.06)	−0.14 (0.06)	−0.33 (0.12)	−0.35 (0.14)
Tonic ERL2	0.18 (0.12)	−0.01 (0.10)	0.20 (0.08)	−0.22 (0.08)	−0.37 (0.15)	−0.34 (0.17)
Alpha asymmetry	0.02 (0.04)	0.02 (0.02)	0.01 (0.02)	−0.09 (0.04)	−0.17 (0.06)	−0.11 (0.05)

The phasic ERL did not vary with task load, *F*(2, 28) = 0.55, *p* > .2 but with type of fixation, *F*(1,14) = 13.09, *p* = .003, adj *η*_p_^2^ = 0.45, being larger with outward than inward saccades. No interaction was observed, *F*(2,28) = 2.31, *p* = .13, adj *η*_p_^2^ = 0.08.

While onset-related activity depicts the effects on input driven information processing, some effects were observed in preparation for a saccade, reflecting anticipatory mechanisms that appear to be driven by attempts to organize incoming information.

At fronto-central leads, a CNV (see Fig. 4 middle raw and Table 4) was observed that increased with task load, *F*(2,28) = 4.45, *p* = .031, adj *η*_p_^2^ = 0.19. Its amplitude was larger for inwards saccades, *F*(1,14) = 19.27, *p* < .001, adj *η*_p_^2^ = 0.55. No interaction between these two factors was found, *F*(2,28) = 0.21, *p* > .2.

Additionally, a slowly increasing asymmetry (tonic ERL) exhibited a task load effect in a time window between 400 and 200 ms preceding saccade onset, *F*(2,28) = 5.39, *p* = .024, adj *η*_p_^2^ = 0.23. This component was also more pronounced for inwards saccades, *F*(1,14) = 12.51, *p* = .003, adj *η*_p_^2^ = 0.43. The interaction reached no significance, *F*(2,28) = 1.06, *p* > .2.

In a later time window, immediately preceding saccade onset (−200 to −50 ms), the task load effect lost significance, *F*(2,28) = 2.43, *p* = .12, adj *η*_p_^2^ = 0.09, while the effect of saccade type remained, *F*(1,14) = 9.72, *p* = .008, adj *η*_p_^2^ = 0.37. There was no interaction, *F*(2,28) = 0.36, *p* > .2.

**Fig. 5:**
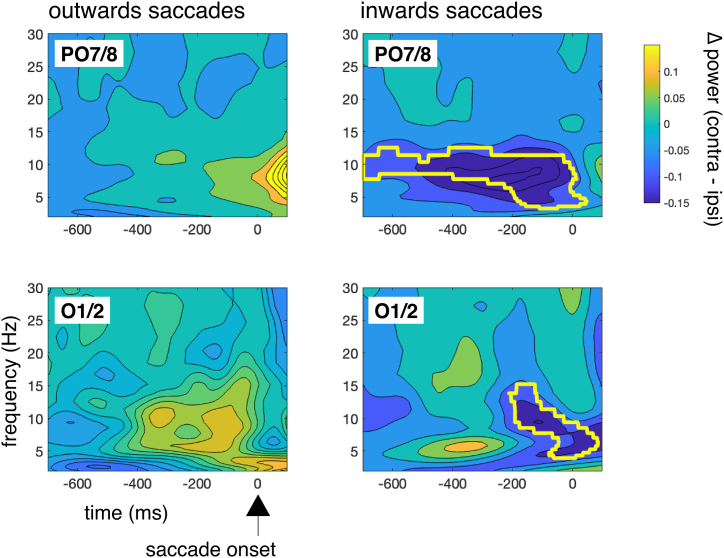
ERSPs and the outcome of the cluster-based permutation test against zero (depicted as the highlighted regions) for the contra-ipsi differences at PO7/PO8 (parietally) and O1/O2 (occipitally). While there were no significant differences for outward saccades in either the parietal or occipital region, inwards saccades demonstrated a sustained suppression of alpha activity contralateral to the direction of the saccade. These findings suggest a higher involvement of covert attentional processes – measured as suppression in alpha power – before the initiation of the saccade.

Alpha asymmetry (see Fig. 5) did not vary with task load, *F*(2,28) = 1.20, *p* > .2. The cluster-based permutation test was supported by an effect of type of saccade, *F*(1,14) = 10.37, *p* = .006, adj *η*_p_^2^ = 0.38. There was no interaction, *F*(2,28) = 0.75 *p* > .2.

## 4 Discussion

In the present study, we classified road sections of a naturalistic driving simulator task into three mental load categories. Based on the load classifications, we investigated the effect of cognitive load on information processing and attentional allocation using eye-activity related EEG activity. A particular interest was to evaluate aspects of mental load not as a merely sustained state of the driver, but as a continuously changing condition that strongly depends on the driving situation and its current demands. Therefore, we conducted two types of analyses. First, we estimated situational task load based on a continuous transformation of Theta and Alpha activity in the EEG, as found in our previous studies [4,5]. Secondly, eye activity-related potentials were used to investigate specific stages of information processing and the distribution of attention within sections of a given task load.

**Fig. 6:**
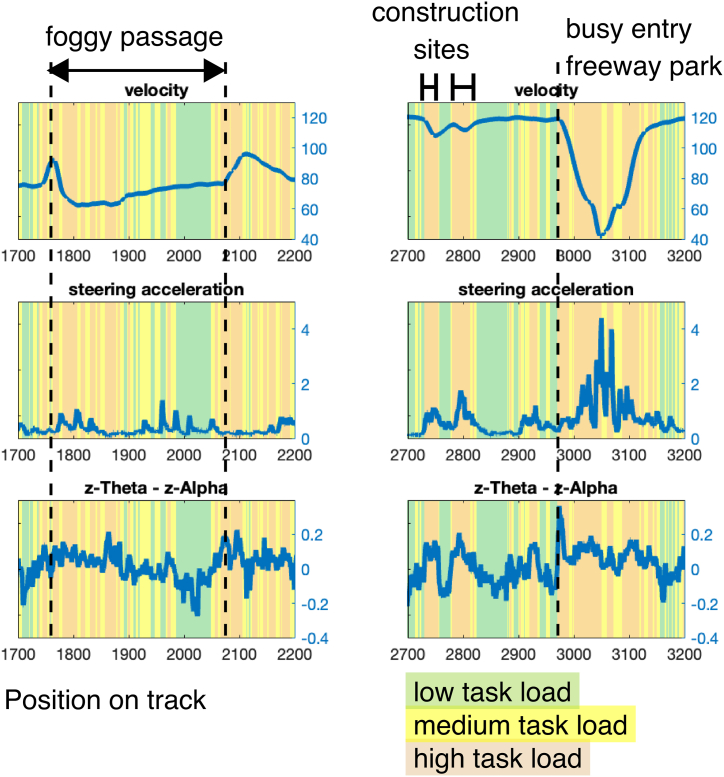
Examples of task load assignments in two particular driving segments (color-coded: red = high, yellow = medium, green = low task load), basic driving parameters (driving velocity and steering acceleration, upper and center plots), and z-transformed theta – alpha activity (which was the EEG basis for task load categorization, lower plots). In the left column, the entry and exit of a foggy passage are depicted. Task load is high when entering the fog and drivers decelerate. While passing the foggy part, the task load continuously decreases while drivers accelerate. In the right column is a short section of the freeway, with two construction sites without a speed limit but needing to decelerate before drivers are directed to a freeway rest area with a quite busy entry. All these distracting events locally increase the task load. When decelerating and leaving the freeway, where drivers additionally drive up a construction vehicle, theta – alpha activity shows a distinct maximum. (For interpretation of the references to color in this figure legend, the reader is referred to the Web version of this article.)

Apparently, the categorization of different task load levels along the driving route by using EEG frequency measures in high temporal resolution revealed plausible results. Following this EEG-based task load categorization, vehicle velocity, and steering activity depicted comparable trends to a similar study with a high sample size [4]. The segments that were assessed to involve the lowest mental load by means of the EEG activity were composed of situations with little steering activity involved, most often found on freeway sections with little traffic – therefore also showing high velocity values. Demanding situations, like passing a construction area or driving through a foggy passage (see also Fig. 6), were reliably detected by the algorithm and categorized as high task load situations, also comparable to our previous findings [4]. For example, the foggy road passage showed an initial increase in task load that continuously declined, accompanied by steadily increasing acceleration. Overall, these findings demonstrate the close link between driver behavior and electrophysiological measures, here particularly the ratio of Alpha and Theta activity, which is in line with previous studies. Since reduced Alpha activity is typically associated with states of increased mental workload and attentional effort e.g. [[24], [31]], and increased Theta activity coincides with mental processing demands and task engagement[e.g [[22], [23]], the ratio of Alpha and Theta activity is especially suitable for reliably representing driver states under natural conditions and the dynamic allocation of cognitive resources along the route see also [32].

The analysis of the blink rate and the saccades also revealed a clear dependence on the current task demand of a situation. Especially in passages classified as difficult, the blink rate decreased, while the number of saccades increased. An increase of saccades (especially outward saccades) in complex situations like city driving compared to less demanding situations like highway driving seems plausible, since in the former case much more visual stimuli at the roadside had to be attended to. Previous studies on the relationship between blink rate and task difficulty, on the other hand, have yielded partly contradictory findings: While some work found a decrease in blink rate with increasing difficulty, just as in our task [33,34], other work yielded opposite findings [35]. As Niezgoda and co-workers [36] state, this relation might strongly depend on the actual task and experimental setting. The suppression of blinks with increasing task difficulty is more likely to be observed in situations where subjects must strongly concentrate on visual stimuli, such as driving in a complex environment. Combined with the driving behavior, eye behavior results can be interpreted as a proof-of-concept that the EEG-based categorization of the driving track is valid and replicable. As we did not include time-domain EEG data nor blink activity for classification, an analysis of eye-activity related EEG activity is sensible and does not involve circular reasoning.

Regarding the analysis of event-related cognitive activity, the use of blinks as event-markers for mental load evaluation has proven beneficial recently. Previously, we have shown that eye-blink-related potentials (bERPs) of the EEG are sensitive to visual demands of natural behavior [7]. Moreover, bERPs are also found to be modulated by higher order cognitive demands like navigation [9]. In this study, modulations of bERPs showed basically the same directions as evoked by motor load, but only the N1 showed a significant decrease in amplitude with increasing task load. This effect has been interpreted as a correlate of attentional narrowing when the task at hand becomes more challenging [7]. The same interpretation may be true in the present driving task, in which a decrease in blink-related N1 has been found with increasing task demands.

Regarding the fixation-evoked ERPs, we observed a quite characteristic pattern that depended on the task load and type of saccade. In particular, N1 and N2 amplitudes decreased with increasing task load. P1, N1, and N2 amplitudes were larger with outward than inward saccades. Interestingly, P1 and P2 increased with increasing task demands, but only for inward saccades. This raises the question of how the two saccade types differ in a driving situation, or – more precisely – what the functional difference between the two types of saccades might be. Regarding a driving task, the essential events should mainly occur in front of the driver, while less driving-relevant events in the periphery (i.e., deviating from the driving direction). It can therefore be assumed that an outward saccade was triggered rather reflexively by a stimulus or an event in the periphery, that is, in a bottom-up fashion. In contrast, an inward saccade (i.e., back to the section of road ahead of the driver) should rather be consciously controlled, that is, planned and executed in a top-down fashion.

There is indeed evidence in our results that outward saccades are rather reflexive. None of the EEG indicators for attentional preparation (i.e., CNV, slow ERL, Alpha suppression) was pronounced for this type of eye movement, while the upcoming fixation evoked a phasic ERL, indicated that spatial signals were processed. Also, P1 and N1, both potentials associated with early, more stimulus-based processing, are more pronounced on outwards than on inwards saccades. Accordingly, a greater P1 has also been observed when regions of higher quantity and relevance of information were presented [14]. For inwards saccades, on the contrary, sustained preparation was observed both in slowly raising CNV and asymmetries that show increased negativity contralateral to the target of the saccade. Similarly, Alpha activity was continuously suppressed contralaterally to the direction of an inward saccade, indicating that the target of the saccade was attended to continuously. Although we did not record eye-tracking data, it appears plausible that inward saccades mainly returned to the center of the vehicle. Thus, inward saccades lead, in most cases, to a renewed fixation of the road. In particular, the Alpha asymmetry indicates that attention is never taken off the road.

In addition to the early P1–N1 complex, task load modulated the fronto-central N2 of the fERPs, being decreased in amplitude when the task became harder, as it was previously observed for bERPs during walking. Moreover, the N2 was larger for outward saccades. The N2 is typically associated with (often conflict-related) cognitive control processes [37,38], for example, as required in the flanker task. Here, the N2 was found to be larger the more executive control was needed to manage the conflicting situation [39]. An increased conflict potential should also be associated with outward saccades, namely when peripheral stimuli distract the driver's attention from the driving-relevant events on the road.

### 4.1 Limitations

The presented data are plausible, but at some points, they still show some weaknesses, which do not yet allow a final assessment. Basically, such realistic driving scenarios lack temporally precise events for the time locking of the EEG. Eye events can be used to keep the situation naturalistic and without experimental interference while being able to have a substantial number of events necessary for the computation of an ERP. Though having the reputation to be a mere workaround, a number of recent studies showed both the reliability and validity of eye-behavior and its neurophysiological correlate in event-related potentials [7,8,40]. Unfortunately, both in-depth eye movement data and lane keeping data were missing, which would allow a local assessment of driving performance. Also, the sample size of the investigated group was rather small, nonetheless high effect sizes were obtained. Adding to that point, the results of the EEG-based categorization of task load and its relation to driving speed and steering variability correspond to those of a previous study with many more subjects [4]. Finally, although the large number of neurocognitive variables considered and analyzed here provide a fairly consistent picture, the contributions of the individual measures to a robust and overarching indicator of driver states need to be further explored in future studies. Therefore, this manuscript is meant to be a conceptual take on the objective assessment of driver perception and attention without an additional task within naturalistic environments.

## 5 Conclusion

The results show that eye-event-related EEG activity is very sensitive to task load in a naturalistic driving task and represents a temporally highly resolved image of specific cognitive processes during driving. For the future, one can imagine that a combination of the parameters presented here could form the basis of a valid driver warning system that goes far beyond a purely frequency-based analysis of the EEG as it has been common for many years. By using events inherent to human behavior, EEG is a tool that enables us to look at attentional allocation in high temporal precision, even when attention is covered and without the involvement of a behavioral driving component, making this procedure very valuable for driving safety research. Blink- and fixation-related potentials might be a promising tool to evaluate *(a)* the overall attentional resources (fERP: lambda, bERP: N2, P3), and *(b)* the spatial allocation of attention (sERP: ERLs, both phasic and tonic). These measures might be used to investigate safety-critical events for drivers in different cognitive states (drowsiness, aging) and how well they manage to allocate visual attention in a manner to anticipate and avert upcoming hazards while driving. While other assessments only involved eye activity measures, using EEG can help to understand the (attentional) motivation behind eye behavior and how much attention can be used to assess the upcoming situation – even preceding eye behavior itself. Especially, the time course of processing safety-critical events can be investigated, leading to new insights into what factors lead to the occurrence of driving errors. Finally, countermeasures can be developed on the premise of considering shifts in attention detected by the means of EEG correlates and therefore increase overall driving safety.

## Declarations

### Author contribution statement

Edmund Wascher: Conceived and designed the experiments; Analyzed and interpreted the data; Wrote the paper.

Emad Alyan: Analyzed and interpreted the data.

Melanie Karthaus: Conceived and designed the experiments.

Stephan Getzmann: Conceived and designed the experiments; Wrote the paper.

Stefan Arnau: Analyzed and interpreted the data; Wrote the paper.

Julian Elias Reiser: Performed the experiments; Analyzed and interpreted the data; Wrote the paper.

### Data availability statement

Data associated with this study has been deposited at https://osf.io/gwnuy/?view_only=3f6e9048570a460da431a2982bfe12d8.

## Declaration of competing interest

The authors declare that they have no known competing financial interests or personal relationships that could have appeared to influence the work reported in this paper.
