# Supplementary material for: Tracking drivers’ minds: Continuous evaluation of mental load and cognitive processing in a realistic driving simulator scenario by means of the EEG
Source: Heliyon. 2023 Jul 3;9(7):e17904. doi: 10.1016/j.heliyon.2023.e17904 (PMC10395282; doi:10.1016/j.heliyon.2023.e17904)
Supplement: Multimedia component 1 [file mmc1.pdf]

LEIBNIZ-INSTITUT
FÜR ARBEITSFORSCHUNG
AN DER TU DORTMUND

Mitglied der Leibniz-Gemeinschaft

IfADo | Ardeystraße 67 | D-44139 Dortmund

Prof. em. Dr. med. A.W. Rettenmeier
Vorsitzender Ethikkommission

Ardeystraße 67
D-44139 Dortmund

Telefon +49. 0172. 3080412
Tel +49. 231. 1084-302 (M. Widynski)
Fax +49. 231. 1084-340
E-mail albert.rettenmeier@uni-due.de

www.ifado.de

Herrn
PD Dr. phil. Stephan Getzmann

- im Hause -

24.03.2017

Ethikantrag zum Projekt „Die Entwicklung verkehrssicherheitsrelevanter Personenmerkmale von Seniorinnen und Senioren und ihre Einflussfaktoren: Eine Längsschnittstudie über fünf Jahre“ (DoBolSiS)

Sehr geehrter Herr Dr. Getzmann,

die Ethikkommission des IfADo hat den Ethikantrag zu Ihrem oben genannten Forschungsprojekt eingehend geprüft und nach Einreichung ergänzender Unterlagen einstimmig befürwortet.

Die Ethikkommission bittet darum, ihr den Abschluss der Studie mitzuteilen.

Im Namen der Ethikkommission wünsche ich Ihnen für Ihr Forschungsvorhaben viel Erfolg.

Mit freundlichen Grüßen

i. a. Rettenmeier

Prof. Dr. A. W. Rettenmeier
Vorsitzender der Ethikkommission

Leibniz-Institut für Arbeitsforschung an der TU Dortmund
Wissenschaftliche Direktoren: Univ.-Prof. Dr. Jan G. Hengstler, Univ.-Prof. Dr. Michael Nitsche, Univ.-Prof. Dr. Edmund Wascher, Univ.-Prof. Dr. Carsten Watzl
Kaufmännischer Direktor: N.N. | Rechtsträger: Forschungsgesellschaft für Arbeitsphysiologie und Arbeitsschutz e.V.
Bank: Helaba | IBAN: DE92 3005 0000 0001 1164 41 | BIC: WELADEDD | VR 2255 beim Amtsgericht Dortmund
